# Mad. Boivin on Supposed Rheumatic Affections of Females

**Published:** 1831-01-01

**Authors:** 


### LXIII.

### OBSERVATIONS ON SOME CASES OF SUPPOSED RHEUMATIC AFFECTION OF THE LOWER EXTREMITIES IN FEMALES. By MADAME BOIVIN.

IT will be some time before we see such minute pathological investigations from the pen of a female writer in this country, as Madame B. occasionally sends forth into the medical world! But we shall let the lady speak for herself.

*Case* 1. A female, 55 years of age, the mother of seven children, had always enjoyed good health till the age of 52, when she was seized with pains in the iliac regions, and in the lower extremities. The catamenia had been suppressed some time previously. Leeches, frictions, anodynes, warm baths, and various other means were used without any relief. The menses returned; but the pains persisted. They were considered rheumatic. A discharge of blood now took place from the uterus, and continued more or less, for many months, without exciting any suspicions in the mind of the medical attendant, till Madame Boivin and M. Dubois were consulted, when they discovered an enormous cancer of the uterus, together with an ovarian disease causing adhesions about the pelvis, which no doubt gave origin to the pains considered rheumatic. She was sent from the MAISON DE SANTÉ into the country to die.

*Case* 2. This case shews the danger of a too speedy return to occupations after an accouchement.

A female did not menstruate till the age of twenty; but after that period had the catamenia regularly continued till her 29th year, when she became pregnant, and went into the hospice of the Ecole de Medecine for accouchement. Her delivery was rapid, the labour only continuing one hour. On the *third* day the female returned to her master's house, and took to her usual avocations of cooking! But she was soon seized with violent fever, swellings of the mammæ, pains in the abdomen, in the left iliac region, to which leeches and fomentations were applied. She was sent into the hospital Necker, where she was properly treated, and comparatively cured; but still there remained a tumour in the left iliac region, with pains in the hip and thigh, which were considered rheumatic by her subsequent medical attendants. She ultimately came under Madame Boivin's observation in a MAISON DE SANTÉ, and on accurate examination, the os uteri was found to be thrown out of its proper position; to be hard and enlarged; while a tumour was felt occupying the iliac region, which was considered as a diseased ovarium. By proper treatment the symptoms were much mitigated; but she was discharged uncured.

Several other cases are related by Madame Boivin, to shew how frequently medical men confound the pains resulting from organic disease in the pelvis, with rheumatic pains of the lower extremities. These we need not detail. The above are sufficient to put practitioners on their guard.—*Journ. Complement.*

